# Grain Structure Evolution of Al–Cu Alloys in Powder Bed Fusion with Laser Beam for Excellent Mechanical Properties

**DOI:** 10.3390/ma13010082

**Published:** 2019-12-23

**Authors:** Michael Rasch, Johannes Heberle, Maximilian A. Dechet, Dominic Bartels, Martin R. Gotterbarm, Lukas Klein, Andrey Gorunov, Jochen Schmidt, Carolin Körner, Wolfgang Peukert, Michael Schmidt

**Affiliations:** 1 Department of Mechanical Engineering, Institute of Photonic Technologies, 91052 Erlangen, Germany; dominic.bartels@lpt.uni-erlangen.de (D.B.); lukas.klein52@gmx.de (L.K.); michael.schmidt@lpt.uni-erlangen.de (M.S.); 2 Bayerisches Laserzentrum GmbH, 91052 Erlangen, Germany; j.heberle@blz.org; 3 Department of Chemical and Biological Engineering, Institute of Particle Technology, 91058 Erlangen, Germany; maximilian.dechet@fau.de (M.A.D.); jochen.schmidt@fau.de (J.S.); wolfgang.peukert@fau.de (W.P.); 4 Department of Material Science and Engineering, Chair of Materials Science and Engineering for Metals, 91052 Erlangen, Germany; martin.gotterbarm@fau.de (M.R.G.); carolin.koerner@fau.de (C.K.); 5 Kazan National Research Technical University, 420111 Kazan, Russia; gorunow.andrej@yandex.ru; 6 Collaborative Research Center CRC 814 – Additive Manufacturing, 91058 Erlangen, Germany; 7 Erlangen Graduate School in Advanced Optical Technologies (SAOT), 91052 Erlangen, Germany

**Keywords:** Powder Bed Fusion with Laser Beam, aluminum-copper wrought alloy, solidification conditions, hot cracking sensitivity, grain structure evolution

## Abstract

Powder Bed Fusion with Laser Beam of Metals (PBF-LB/M) is one of the fastest growing technology branches. More and more metallic alloys are being qualified, but processing of aluminum wrought alloys without cracks and defects is still challenging. It has already been shown that small parts with low residual porosity can be produced. However, suffering from microscopic hot cracks, the fracture behavior has been rather brittle. In this paper different combinations of temperature gradients and solidification rates are used to achieve specific solidification conditions in order to influence the resulting microstructure, as well as internal stresses. By this approach it could be shown that EN AW-2024, an aluminum-copper wrought alloy, is processable via PBF-LB/M fully dense and crack-free with outstanding material properties, exceeding those reported for commonly manufactured EN AW-2024 after T4 heat treatment.

## 1. Introduction

Modern additive manufacturing (AM) has its origin in the invention of the first stereolithography machine by Charles W. Hull in 1984 [1]. Since then AM has drawn increasing interest of both science and industry [2]. New technologies not only for the processing of polymeric but also metallic materials were developed. The most important process for additive manufacturing of metals is currently powder bed fusion with laser beam [3] abbreviated as (PBF-LB), according to the nomenclature given in DIN EN ISO/ASTM 52900 [4]. To date, several alternative terms are being used for naming this process due to the past lack of international standardization. Direct Metal Laser Sintering (DMLS), Selective Laser Melting (SLM), Laser Cusing, Laser Fusing and Laser Beam Melting (LBM) all describe the same methodology of using a laser source and a powder bed for the fabrication of three-dimensional metallic parts.

### 1.1. PBF-LB/M and Used Materials

**Figure 1:**
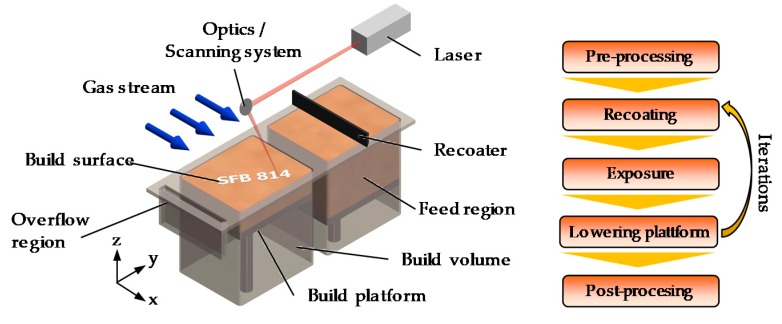
Left: Schematic drawing of a PBF-LB/M machine. Right: Build cycle.

Powder bed fusion with laser beam of metals (PBF-LB/M), is described according to ISO 17296-2 as a process where a heat source selectively melts a powder layer by layer [5]. This allows tool-free manufacturing of highly complex parts. Fine metallic powder with a typical particle size of 1 µm to 65 µm is deposited layer-wise on a build platform (see Figure 1).

An energy source, a laser in the case of PBF-LB/M, melts the deposited powder layer selectively. After each exposure the build platform is lowered by a small distance and a new layer of powder is deposited. By adding millions of scan tracks together, a part is formed. The application of a laser source for processing typically leads to rapid cooling and high temperature gradients in the range of (7 × 10^4^)–(4 × 10^7^) K/s and (5 × 10^4^)–(5 × 10^6^) K/m [6,7], respectively, compared to conventional manufacturing processes such as casting and forming. This thermal load leads to high stresses during solidification and cooling of the final component. Therefore, this process is sensitive to hot and cold cracking. As AM of metals is a very young technology, only a limited choice of materials are qualified for PBF-LB/M. Commercially available aluminum alloys are generally derived from conventional cast alloys based on aluminum and silicon [8]. Exceptions for this are specially developed alloys for PBF-LB/M such as e.g., Scalmalloy^®^, which are characterized by a higher strength [9]. However, these alloys are typically either lacking high strength and ductility or consist of rare elements like scandium, which increases the costs per kilogram excessively [10]. Consequently, the qualification of aluminum alloys used for forging such as alloys of series 2xxx is considered as mandatory [11].

### 1.2. Al–Cu Alloys in PBF-LB/M

First processing of Al-Cu alloys EN AW-2022 and 2024 with relative densities close to 100% in PBF-LB/M was reported in 2014 [12], followed by ENAW-2219 and 2618A later in the same year [13]. Further investigations on the mechanical properties of additively manufactured EN-AW 2618A have shown appropriate values for elongation for vertically built and heat-treated samples within the range of the conventional material [8]. However, horizontally manufactured specimens are characterized by a brittle cracking behavior, indicating the presence of micro hot cracks. Analysis of the chemical composition of the fabricated samples and raw material showed deviations, too. For EN AW-2219 specimens manufactured in PBF-LB/M, elongations almost twice as high as for conventionally fabricated material were observed, but the alloy’s composition was not confirmed [14]. Zhang et al. proved the processability of manufacturing fully dense and crack-free EN AW-2024 samples in 2016 using PBF-LB/M [15]. The study found a critical threshold energy density of 340 J/mm^3^ which needs to be exceeded for allowing a defect-free fabrication. It is stated, that if this energy density is not reached, the backfilling of voids with liquid material is not sufficient, thus supporting the formation of hot cracks. Fabricated samples were subsequently analyzed using tensile testing. No heat treatment was mentioned. However, mechanical properties of as-built specimens fell below the ones of EN AW-2024 in T3, T4 or T6 heat treatment condition but exceeded the values of heat treatment O, which is equivalent to no heat treatment being applied (see Table 1) [16].

Nie et al. conducted micro- and nano-hardness measurements on single track lines and small cubes fabricated using PBF-LB/M [17]. During testing small variations in nano-hardness at weld borders and in the center of the part were identified. These deviations occur due to columnar dendrites at the boundaries and equiaxed grains in the center. Further experiments, based on a study conducted by Zhang et al. [18], have shown the possibility of fabricating crack-free samples by addition of up to 2.5 wt.% zirconium due to grain refinement achieving a mainly globular grain structure. Furthermore, the scanning speed could be increased by factor of 3 without the occurrence of cracks, though reduced relative densities were observed [19]. Tensile testing of the unmodified specimens showed slightly lower yield strength (YS) and ultimate tensile strength (UTS) as commonly manufactured EN AW-2024 in T3 and T6 condition. The addition of Zr leads at 2 wt.% to a maximum of YS of (464 ± 2) MPa and UTS of (493.30 ± 10) MPa. However, key information on heat treatment strategies and building orientation during manufacturing are missing. 

Lopez-Botelle et al. studied the grain growth behavior of PBF-LB/M-manufactured EN AW-2024 using low processing speeds resulting in a predictive model for the estimation of melt pool size, gradient development and average grain size [20].

Further investigations on an aluminum alloy similar to EN AW-2024 (Al–3.5Cu–1.5Mg–1Si) were carried out by Wang et al. for the identification of an appropriate process window for the fabrication of crack-free specimens. Characterizations of the manufactured material have shown similar mechanical properties as conventionally produced samples [21]. In-situ alloying approaches by adding both, larger amounts of copper and TiB_2_ micro particles lead to improved mechanical properties [22]. Copper-enrichment resulted in increased compressive strength, while the addition of up to 5 wt.% TiB_2_ entailed in improved compressive yield strength. Another in-situ alloying approach was carried out by Karg et al. [23]. By mixing four different base powders the alloy composition was created homogeneously in-situ.

### 1.3. Metallurgical Properties of Al–Cu Alloys, Hot Tearing Phenomenon Criterion and Grain Morphology

**Figure 2:**
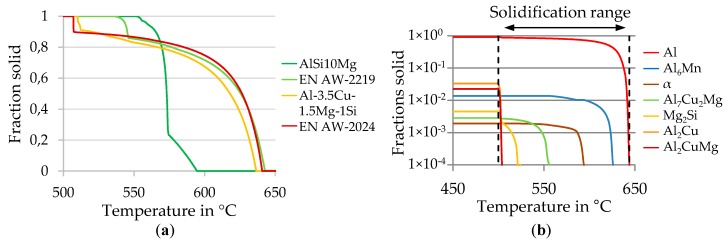
(**a**) Solidification path of different Al–Cu alloys compared to AlSi10Mg (**b**) Phase forming during solidification in EN AW-2024. Calculated with JMatPro 10.4.

Al–Cu-based alloys are characterized by a larger solidification range compared to other aluminum alloys. According to Figure 2a this range is six times larger for EN AW-2024 (134 °C) in contrast to AlSi10Mg (22 °C), thus, allowing phase segregation (see Figure 2b). During solidification the composition of the alloy changes at the solidification front. This supports the formation of either columnar, dendritic or equiaxed grain structures. The corresponding center of the solidifying structure consists of pure aluminum, while the grain boundary and inter-dendritic regions are mainly Al_2_Cu. 

During solidification Al–Cu alloys are exposed to shrinkage and displacement due to thermally introduced shear or tensile stresses [24]. The resulting voids require a backfilling by the remaining liquid volume to avoid hot crack formation. Over the last years, hot cracking susceptibility has been addressed by different researchers and several criterions have been developed to describe this mechanism. At this point the two most common and adapted ones [25] are hereinafter introduced. Clyne and Davies deduced the so-called Cracking Susceptibility Coefficient (CSC) for casting applications in 1975 [26]. They claim that hot cracking is the result of tensile forces on the dendrite arms in the mushy zone. The solidification path is sectioned into two regions; one where backfilling can take place (solid fractions of 10–60%) and one where this is hardly possible due to the low permeability of the dendritic network (solid fractions of 1–10%). If the liquid phase falls below 1%, the dendrite arms will be assumed to be fully coalesced. The time spent in this vulnerable region divided by the time spent outside defines the CSC.

Rappaz, Drezet and Germaud (RDG) addressed the hot cracking sensitivity (HCS) in their work “New Hot-Tearing Criterion” [27]. By removing some simplifications made in the CSC, like the neglection of the deformation of the solid network, this criterion becomes more extensive. Only external forces and localized stresses remain unheeded and the strain rate in the mushy zone is assumed to be uniform. Hot crack formation is stated to appear for pressures exerted to the liquid below a defined cavitation pressure. Thus, the drop in liquid pressure is estimated based on the solidification shrinkage $\Delta p_{sh}$ and deformation $\Delta p_{mec}$ (see Equation (1)) [28]:

(1) $$\Delta p_{sh}\;+\;\Delta p_{mec}\;=\;\frac{180\mu }{G\lambda^{2}}\left[v_{T}\beta A\;+\;\frac{\left(1\;+\;\beta \right)B\dot{\varepsilon }}{G}\right]$$

(2) $$\mathrm{with}\;A\;=\;\int_{T_{co}}^{T_{L}}\frac{f_{s}{\left(T\right)}^{2}}{\left(1\;-\;f_{s}\left(T\right)\right)²}dT\;\mathrm{and}\;B\;=\;\int_{T_{co}}^{T_{L}}\frac{\int f_{s}\left(T\right)dTf_{s}{\left(T\right)}^{2}}{\left(1\;-\;f_{s}\left(T\right)\right)³}dT$$

where *µ* = viscosity of the liquid in the mushy zone, *λ* = distance between the smallest obstacles determining the permeability of the mushy zone (secondary arm spacing for dendrite, distance between columns or diameter of equiaxial grains [29]), $v_{T}$ = solidification speed, β = shrinking factor between liquid and solid, $\dot{\varepsilon }$ = deformation rate,$\;f_{s}$ = fractions solid, $T_{L}$ = liquidus temperature and $T_{co}$ = temperature of coalescence (>0.98 $f_{s}$ or more than 2% eutectic), *G* = temperature gradient.

**Figure 3:**
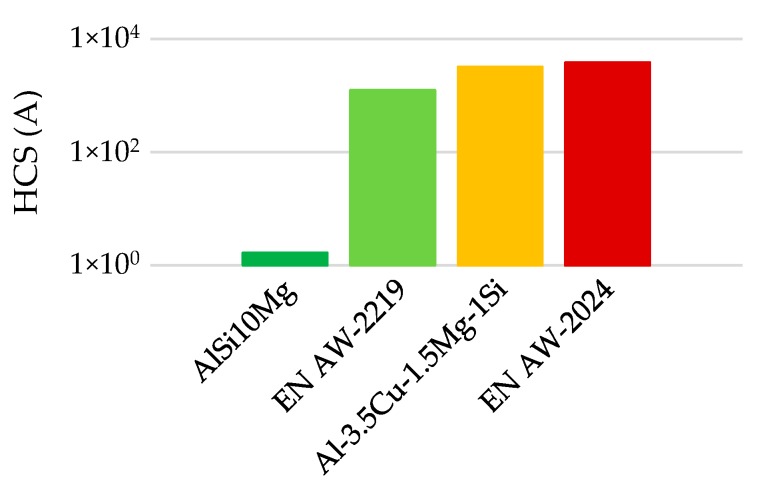
HCS (A) of different aluminum alloys.

In this assumption the calculation is highly dependent on the lower bound of the integral as the determination of $T_{co}$, especially for supercooled liquids, is difficult as the expected and the resulting phase formation might vary. For comparison of HCS of different aluminum alloys, the calculation of factor A is considered to be sufficient as factor B is expected to show a similar impact as factor A does (see Figure 3), but it is harder to calculate. In general, a higher HCS (A) value results in a more crack-sensitive process [28].

The EN AW-2024 alloy is characterized by late formation of eutectics and a huge solidification range, as shown in Figure 2, resulting in the highest HCS amongst aluminum alloys. As integrals A and B depend only on the solidification path, no derivation of recommendations for the reduction of HCS by processing parameters is possible.

Thus, by extending Equation (1) with the cavitation depression $\Delta p_{c}\;$(see Equation (3)), the calculation of the maximum strain rate ${\dot{\varepsilon }}_{p,max}$, that can be sustained by the deepest part of the mush before a void forms, is possible. The HCS index is than defined according to Equation (4) [28].

(3) $$\Delta p_{c}\;=\;\Delta p_{sh}\;+\;\Delta p_{mec}$$

(4) $$HCS\;=\;\frac{1}{{\dot{\varepsilon }}_{p,max}}\;=\;\frac{1\;+\;\beta }{G\cdot \left(\frac{\lambda^{2}}{180}\frac{G}{\mu }\Delta p_{c}\;-\;v_{T}\beta A\right)}B$$

**Figure 4:**
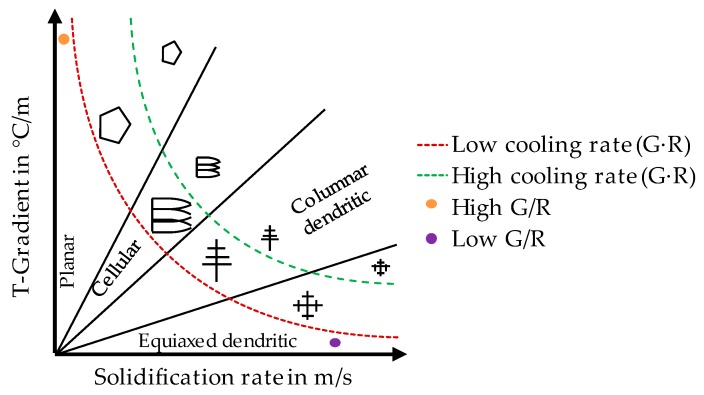
Effect of temperature gradient G and solidification rate R on the morphology and size of the solidification microstructure [30].

Critical influencing factors on HCS are the temperature gradient *G*, the velocity of the isotherm $v_{T}$, also known as solidification rate $R=\frac{\dot{T}}{G}=v_{s}\cdot \cos\alpha$ ($v_{s}$ is welding speed, α the angle of the normal on the solidification front relative to the welding direction [30]), and the obstacle distance *λ*, which describes the offset between two already solidified structures in the melt during solidification. This distance can be calculated for the corresponding grain morphology, e.g., primary arm spacing (PDS) $\lambda_{1}=\frac{a}{{\left(G^{2}xR\right)}^{n}}$ for cellular arms and secondary arm spacing (SAS) $\lambda_{2}=\frac{a}{{\left(GxR\right)}^{n}}$ for columnar dendrites [31,32]. In both cases factors *a* and *n* are constant material parameters. Furthermore, $\lambda_{2}$ is applicable for the grain size of globular equiaxed dendritic grains in a slightly modified RDG [33]. Additionally, knowledge about the ratio G/R and the product G·R is crucial for assessing grain morphology [30] (see Figure 4).

According to Equation (4), higher values for temperature gradient *G*, solidification rate *R* and obstacle distance *λ* are desirable. However, these variables are not independent of each other as e.g., higher cooling rates lead to a higher solidification rate, thus reducing the obstacle distance. Another influencing factor is the transition from cellular to columnar and columnar to equiaxed microstructure as it defines the value used for *λ*—grain size, PDS or SAS. Unfortunately, the exact transition for both is mostly unknown especially for the high G and R in PBF-LB/M. 

### 1.4. Aims of the Work

The correlation between hot cracking tendency and solidification conditions in PBF-LB/M was not addressed in the literature so far. Therefore, the goal of the presented work is to clearly demonstrate the influence of solidification conditions on resulting grain structure, hot crack tendency and final mechanical properties. A combination of experimental studies, simulation and metallurgical analysis is used to obtain generalizable information for processing and tailoring aluminum wrought alloys.

## 2. Materials and Methods

### 2.1. Laser Powder Bed Fusion Machine

All experiments have been carried out on a SLM 280^HL^ (SLM Solution Group AG, Lübeck, Germany). A single mode laser provides a laser power (P_L_) of up to 400 W. For the conducted experiments a minimal spot diameter of $w_{0}\;$= 78 µm and argon as shielding gas with oxygen values below 0.01% are used. The scanning direction is rotated by 37° after each layer. A customized building envelope reduction with an extended platform preheating is installed in order to achieve preheating temperatures of up to a nominal value of 500 °C. The actual temperature on the surface of the substrate plate was determined by thermocouple measurements and is defined as preheating temperature T_pre_ in this study. 5 mm thick substrate plates made of EN AW-5083 with a diameter of 90 mm were used.

### 2.2. Powder Material

**Figure 5:**
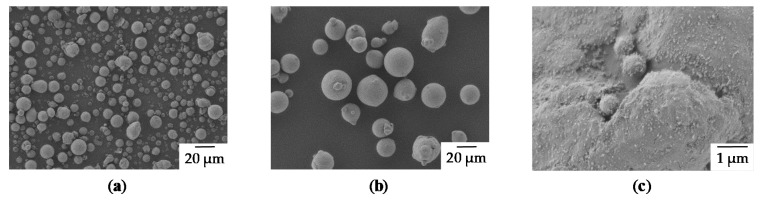
(**a**) Fine particle fraction (EHT = 1 keV, WD = 6 mm, Mag = 500×) (**b**) Corse particle fraction (EHT = 1 keV, WD = 6 mm, Mag = 500×) (**c**) Surface of a particle covered with SiO_x_ (EHT = 1 keV, WD = 5 mm, Mag = 15.000×).

AK 24 [34] bulk material from Otto Fuchs KG (Meinerzhagen, Germany) was atomized by Nanoval GmbH & Co. KG (Berlin, Germany) using argon as processing gas in order to obtain spherical powder particles (see Figure 5). The particle shape and surface morphology were characterized by scanning electron microscopy (SEM) using a Gemini Ultra 55 (Carl Zeiss AG, Oberkochen, Germany) operated at an acceleration voltage (EHT) of 1.0 kV using a SE2 detector and a working distance (WD) of 6 mm.

**Figure 6:**
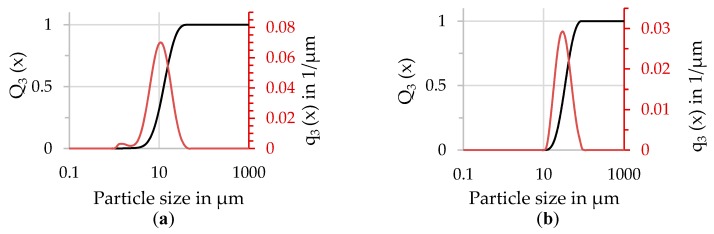
Particle size distribution of (**a**) fine fraction (**b**) coarse fraction. Q_3_ = volume cumulative distribution, q_3_ = volume density distribution.

Subsequently, air classification yielded the finally used powder fractions. Particle size distributions (PSD) of the produced particles were measured by laser diffraction particle sizing (see Figure 6) using a Malvern Mastersizer 2000 equipped with a Scirocco 2000 dry dispersion unit (Malvern Panalytical, Malvern, UK). The applied dispersion gas pressure was set to 2 bar. The two resulting fractions were appropriately labeled according to their volume-median-particle-diameter (d_50.3_) as fine (d_50.3_ = 12.6 µm) and coarse (d_50.3_ = 34 µm).

The chemical composition was measured using inductively coupled plasma atomic emission spectroscopy (ICP-OES) (Optima 8300, Perkin Elmer, Waltham, MA, USA) after digestion of the respective sample in aqueous solutions of aqua regia and hydrofluoric acid. Argon was used as plasma (flow rate 10 L/min) and nebulizer gas (0.6 L/min). The auxiliary gas (0.2 L/min) was N_2_. Calibration and sample solutions were fed to the nebulizer at 3 mL/min. The plasma power was 1500 W and a 6-point calibration was performed in the concentration range between 1 mg/L and 100 mg/L. The relative standard deviation (RSD) of the determined elemental concentrations typically was smaller than 2% (see Table 2). All determined alloying elements are in the defined range for raw powder as well as for the manufactured specimens using PBF-LB/M. The proportion of the elements with a low boiling point is slightly reduced in the PBF-LB/M sample. To improve the flowability, which is especially necessary for the fine fraction due to its poor flow behavior, 0.3 wt.% Aerosil R106 particles were added similar to a protocol described in [35]. Subsequent dry-coating was carried out using a Turbula T2A shaker (Willy A. Bachofen AG, Muttenz, Switzerland) at a speed of 96 rpm and a mixing time of 1 h. Electron imaging has shown a homogeneous distribution of the SiO_2_ particles on the powder surface (see Figure 5c).

### 2.3. Used Parameter Sets

The development of process parameters was carried out in two consecutive steps. In the beginning, four different parameter sets using the coarse powder fraction for layer thicknesses d_s_ of 40 µm and 50 µm were developed aiming to achieve a microstructure formation similar to the regions presented in Figure 4. By modifying preheating temperature T_pre_ and scanning speed v_s,_ varying temperature gradients *G* and solidification rates *R* are expected (see Table 3, “∙” mark the classification compared to the other sets). The hatch distance Δy is kept constant for the set 1 and 2 and varied for the other sets.

The second step focuses on the improvement of the most suitable parameter sets 1 and 4 determined in the first approach in the subsequent sets 5 and 6 (see Section 3.1 for more detailed information). Set 1 showed less to no hot cracks, however porosity related to insufficient melting as well as upcoming keyhole porosity appeared. The reduction of the layer thickness allows lowering the used laser power in order to avoid keyhole porosity while still achieving sufficient melting. Set 4, with respect to the expected solidification conditions has the highest potential to form globular grains but did show short cracks.

After the maximum preheating temperature had to be lowered to T_pre_ = 200 °C due to the previously observed oxidation, a higher gradient is expected. To remain at a low G/R the scanning speed that corresponds to the cooling rate is increased. The layer thickness d_s_ was also reduced, thus supporting sufficient melting. A side effect is the reduction of the molten pool volume, which directly affects the mushy zone volume, further reducing the hot cracking tendency [37]. For all conducted experiment at this step the fine powder fraction was used. The modified sets of parameters are listed in Table 4.

### 2.4. Simulation Setup

**Figure 7:**
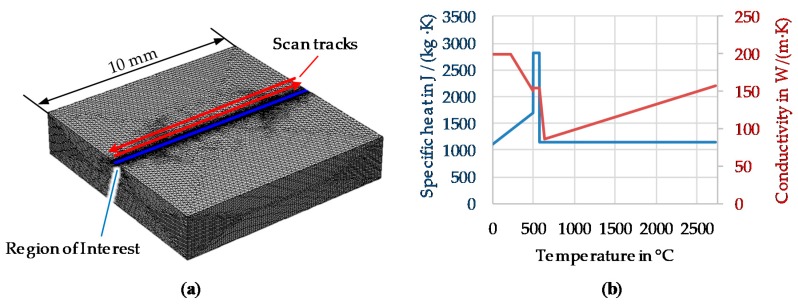
(**a**) Meshed geometry for the simulative setup (**b**) Specific heat capacity and thermal conductivity calculated with JMatPro 10.4 and smoothed.

For the approximate determination of the order of magnitude for the cooling rates and temperature gradients a simple setup in COMSOL Multiphysics 5.4 for a heat conduction model was used, neglecting melt pool flow, internal melt convection, evaporation, the presence of powder and different scan track length due to the scan vector rotation. A cuboid with a size of 10 × 10 × 2 mm³ models the sample cuboid from the experiments. The mesh element size is set globally to 2 × 10^−6^–2 × 10^−4^ m but refined to a maximum of 2 × 10^−5^ m in the region of interest (see Figure 7a) blue region). The boundary conditions of the side and top faces are modeled with convective heat flux from solid to air using COMSOL built-in correlations to match the condition of external natural convection [38]. The bottom has an open boundary condition modelling conductive heat flow to an infinitely large volume. This is equivalent to the condition of a significantly larger base plate of the AM machine on which the cuboid is built on. The laser beam is modeled as a surface heat source on the top surface of the cuboid, with 2D Gaussian intensity distribution (Equation (5) [39]):

(5) $$I\left(x,y\right)\;=\;\left(1\;-\;R_{L}\right)\frac{2P_{L}}{\pi w_{0}^{2}}e^{-2\left(x^{2}\;+\;y^{2}\right)/w_{0}^{2}}$$

where $P_{L}\;$= laser power, $R_{L}\;$= reflectivity, $w_{0}\;$= 1/e² radius of the beam waist (see Section 2.1.)

A surface heat source is reasonable as in aluminum the laser beam is fully absorbed within a few nanometers and keyhole formation was mostly avoided in the experiments; a similar simulative setup with a more complex heat source was already validated for welding of EN AW-2024 by Caiazzo and Alfieri in terms of peak temperature and melt pool depth [40]. The heat source is moved from the center of an edge at the top surface to the edge on the other side and reversing back laterally shifted by the hatch distance Δy.

The implementation of temperature dependent specific heat capacity and thermal conductivity (see Figure 7b, extracted from the Calculation of Phase Diagrams (CalPhaD)-based material database JMatPro 10.4 [41] for the alloy´s composition) allows an approximation of the latent heat of the phase change from solid to liquid [42] and temperature-dependent heat conduction.

**Figure 8:**
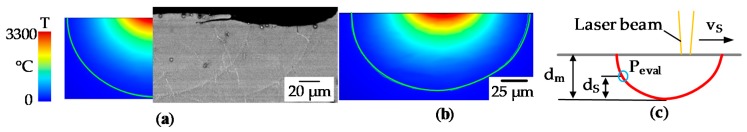
(**a**) Comparison of a cross-section for P_L_ = 133 W, v_s_ = 80 mm/s, Δy = 60 µm, T_pre_ = 0 °C from the simulation (melt pool boundary marked by green line) and experiment (SEM, EHT = 20 kV, WD = 8.6 mm, Mag = 750×, AsB-Detector) (**b**) longitudinal cross-section from simulation (**c**) Evaluation point P_eval_ (blue circle) for the solidification conditions.

The actual reflectivity of the material during the melting process is fairly unknown but has a high impact on the energy input. Therefore, in the simulation the reflectivity was adjusted to fit the experimental results. For this purpose for each parameter set the simulations were carried out under variation of the reflectivity and comparing the molten pool depth *d_m,sim_* to the experimental ones *d_m,ex_* determined at the top layer by averaging the depth of five tracks (see Figure 8a)*_._* From this the reflectivity parameter was adjusted accordingly. Following the approach of Promoppatum et al. [39], who validated this simulation setup for Inconel, the solidification conditions by means of the acting temperature gradient G, solidification rate R, cooling rate dT = G/R and the ratio GxR have been determined at P_eval_ (see Figure 8c) on the second scanning path in the center of the cuboid. In advance simulations with more scanning paths have been conducted but it was found that the solidification conditions do not change much with respect to the assumed contributions of the simplifications made on the actual solidification conditions. P_eval_ is the upmost point on the symmetry plane of the molten pool that will not be remolten in a consecutive layer and therefore due to the epitaxial grain growth mainly responsible for the grain morphology. This point is determined by the intersection of a line parallel to the surface at the melt pool depth d_m_ minus layer thickness d_s_ and the molten pool boundary (see Figure 8c). Furthermore, it is known that hot cracks start to form at the tip of the solidification front, thus showing the high importance of this point.

Simulations were run of a workstation equipped with a 32 cores Ryzen 2990WX CPU, equipped with 3.0 GHz, and 128 GB RAM. 

### 2.5. Characterization

For characterization of the relative density and the microstructure all specimens have been cut in build direction, ground (P240–P1200) and polished with 6 µm diamond suspension followed by a 0.05 µm OP-S finish. Polishing was done with a 30 mm holder, 10 N per holder and for 20 min in both cases. The relative density was determined by analysis of stitched cross-sections obtained with a high-resolution optical microscope, Aristomet (Leica Microsystems GmbH, Wetzlar, Germany), covering the full sections of the specimens with exception of the rough outer edges using of the Otsu-threshold approach [43] and conversion to black and white according to VDI 3405 Blatt 2 [44]. The microstructure of the specimens was investigated either by etching the cross-section with Gnibel´s reagent or by using a scanning electron microscope equipped with an energy dispersive X-ray (EDS), electron backscatter diffraction (EBSD) and angular selective backscattered electron (AsB) detectors (Zeiss Merlin). The mean grain width was determined by the number (N) of grains that are crossed by a line of 3 mm length perpendicular to the built direction and averaged over 3 measurements (3 mm/N). For mechanical testing, half of the tensile samples have been exposed to a T4 heat treatment. Samples were annealed at a temperature of 500 °C in a furnace of type N11/HR (Nabertherm GmbH, Lilienthal, Germany) for 3 h and subsequently quenched in water, before being aged at room temperature for a minimum of 14 days. Vertical specimens have been milled according to DIN 50125—B 6 × 30 and horizontal samples according to DIN 50125—B 8 × 40. The tensile testing was measured by Hirschvogel Holding AG (Denklingen, Germany) according to ISO 6892-1 at room temperature [45].

## 3. Results and Discussion

### 3.1. Process Window

**Figure 9:**
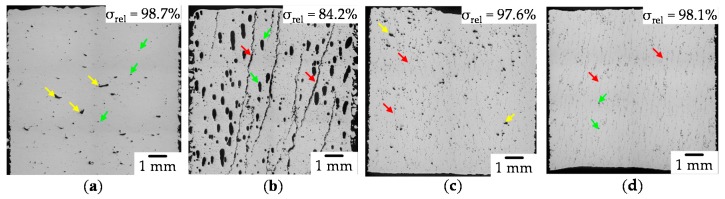
Cross-section (optical microscopy) of different specimens with the highest relative density of set 1–4 (**a**) P_L_ = 200 W, v_s_ = 83.3 mm/s, Δy = 70 µm, T_pre_ = 20 °C (**b**) P_L_ = 200 W, v_s_ = 83.3 mm/s, Δy = 70 µm, T_pre_ = 350 °C (**c**) P_L_ = 300 W, v_s_ = 1240 mm/s, Δy = 78 µm, T_pre_ = 20 °C K (**d**) P_L_ = 400 W, v_s_ = 2820 mm/s, Δy = 83 µm, T_pre_ = 300 °C. Yellow arrows mark insufficient melting, green gas porosity and red hot cracks.

In the first step, only low relative density could be achieved. The cross-sections of the specimens obtained with optical microscopy with the highest relative density of each set are shown in Figure 9.

The cross-section typical for Set 1 (see Figure 9a) shows a mixture of insufficient melting (yellow arrows) and upcoming gas porosity due to keyhole instabilities [46] (green arrows). Therefore, further increase of the line energy density would not increase the relative density. The low relative density at this set is unexpected because Zhang et al. [15] reported relative densities of up to 99.8% for this process window. However, as only few hot cracks could be found in the sample, this set of parameters is considered for further investigation. Set 2 (see Figure 9b) is characterized by huge porosity due to overheating (blue arrows) and hot crack formation (red arrow) throughout the complete specimen. It appears that once a gas pore is formed, the heat flow might be hindered in the subsequent layer and a new gas pore is formed most likely at the same spot. The number of hot cracks is comparably low, but these defects grow throughout the complete specimen. If once a crack is formed, obviously it acts as local stress accumulator allowing the crack to grow extensively, even if the tension on the liquid film surrounding the grain is expected rather weak due to the low gradient. The long time spent in the vulnerable region (see Section 1.3.) supports the crack formation as well. A reduction in line energy density would help to reduce gas pore formation, however, no reduction in hot crack tendency is expected. Furthermore, the preheating temperature had an impact on the deposited powder as long processing times in combination with residual oxygen in the atmosphere resulted in an increase of the oxide layer. This is indicated by a change in color and the formation of a so-called sinter cake. Parameter set 3 (see Figure 9c) is characterized by regions, which were melted insufficiently (yellow arrows) and short hot cracks (red arrows). Set 4 led (see Figure 9d) to a small amount of gas porosity (green arrows) and micro hot cracks tending to spread from one pore to another (red arrows). Additionally, high preheating temperatures resulted in oxidation of the powder. In general, it was observed that high scanning speeds in addition to preheating result in reduced crack length. Further reduction in scanning speed, while also omitting the preheating of the build platform supports the fabrication of crack-free specimens. Based on these findings, further experiments with a reduced layer thickness of 20 µm and preheating temperatures aiming at improving the quality of the parts were conducted. Additionally, the smaller layer thickness allows to reduce the size of the molten pool and at the same time the size of the mushy zone resulting into a lower HCS [37].

**Figure 10:**
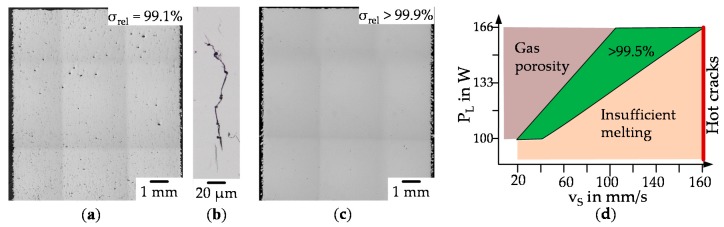
Cross-section (optical microcopy) of different specimens with the highest relative density of set 5 and 6 (**a**) P_L_ = 400 W, vs = 6500 mm/s, Δy = 20 µm, T_pre_ = 200 °C (**b**) magnification of a hot crack in set 5 (**c**) P_L_ = 133 W, vs = 80 mm/s, Δy = 60 µm, T_pre_ = 20 °C (**d**) process window for crack free manufacturing.

Figure 10 illustrates the results of the second iteration step for the identification of process parameters. Set 5 (see a) shows that samples with relative densities >99% could be obtained. Smaller pores were detected, which could be attributed to insufficient melting. The size of the micro hot cracks appears smaller compared to Figure 9c,d with a width below 1 µm (Figure 10b). Specimens fabricated with those process parameters are for sure not suitable for loaded components but the volume built per second is quite high. A subsequent hot isostatic pressing might heal the cracks creating useable parts. Set 6 (Figure 10c) finally includes samples with negligible residual porosity. The complete process window is shown in Figure 10d. A scanning speed of 160 mm/s seems to be a hard border for the appearance of hot cracks. At a hatch distance of 60 µm gas porosity is clearly visible, further decreasing for hatch distances of up to 80 µm and for even higher distances hot cracks start to form. It appears that the local temperature gradient is lowered by narrow hatching wherby cracks can be avoided.

### 3.2. Simulation Results

The solidification conditions have been calculated according to Section 2.4 (see Table 5). The sample numbers used are the same as the figure numbers in Appendix A and are referred to in the following section for discussion. The different values for the reflectivity R_L_ can be attributed to the change in process dynamics, mainly due energy extraction due spatter formation at higher scanning speeds and although due to stronger evaporation only forming a pseudo keyhole not allowing double reflection due to the higher used intensity [47]. 

### 3.3. Microstructure Characterization

**Figure 11:**
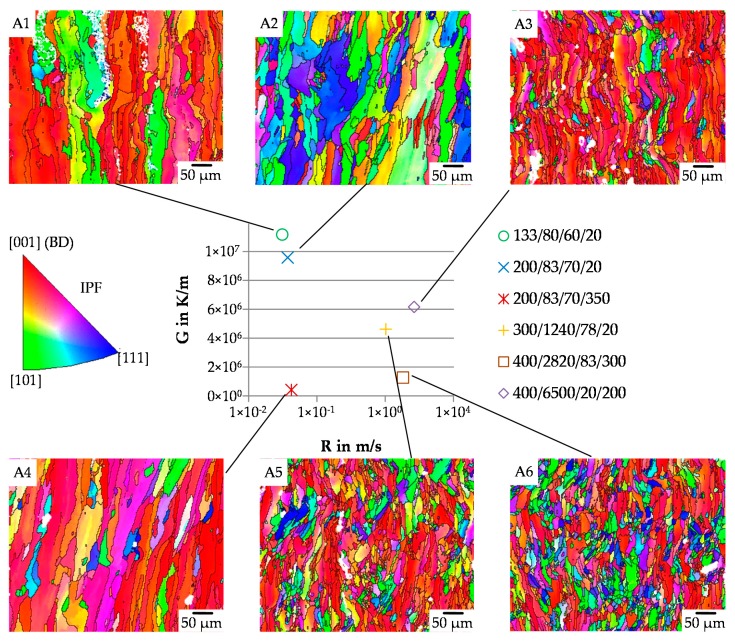
Characteristic grain structure in dependence of G and R. For enlarged EBSD-pictures with included pole-figures see Figure A1, Figure A2, Figure A3, Figure A4, Figure A5 and Figure A6. Nomenclature of the legend: (P_L_/v_S_)/(Δy/T_pre_). Grain orientation is according to IPF coloring. White regions mark “no detection”.

Depending on the processing parameters, different microstructures evolve (see Figure 11). According to Figure 4 [29] a higher cooling rate (G × R) assumingly leads to a finer grain structure whereas a change in G/R significantly changes the morphology. The recognizable epitaxial grain growth in [100] direction [48,49], which can be found throughout every sample, is typical for PBF-LB/M process.

**Figure A1:**
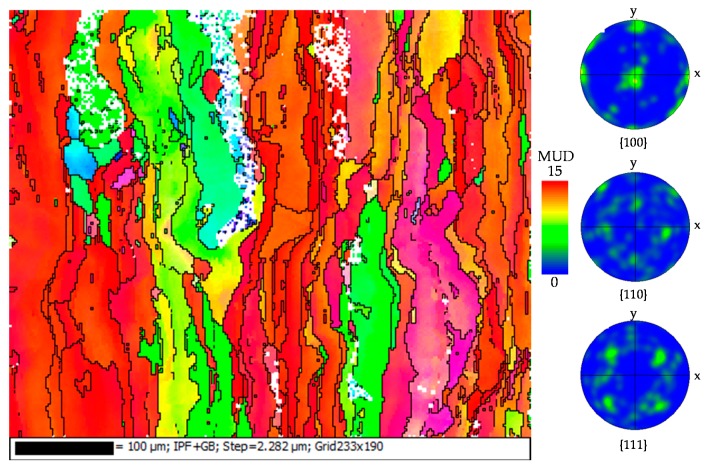
EBSD picture and pole figures for P_L_ = 133 W, v_s_ = 80 mm/s, Δy = 60 µm, T_pre_ = 0 °C.

**Figure A2:**
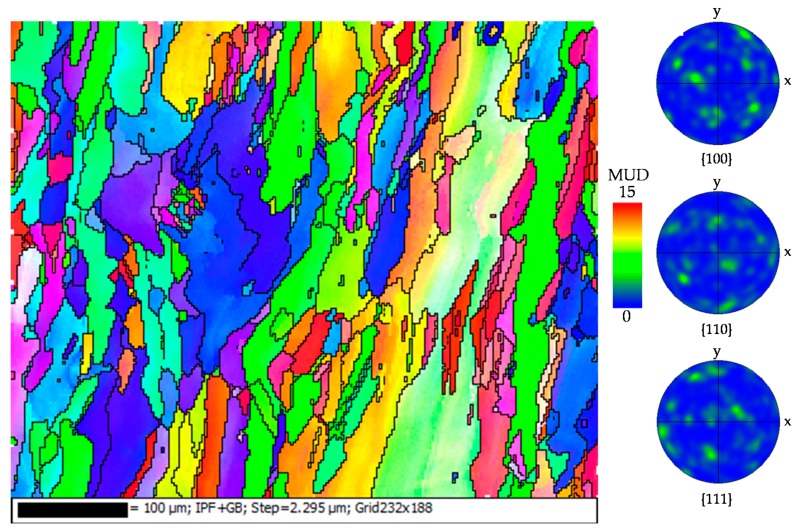
EBSD picture and pole figures for P_L_ = 200 W, v_s_ = 83.3 mm/s, Δy = 70 µm, T_pre_ = 0 °C.

**Figure A3:**
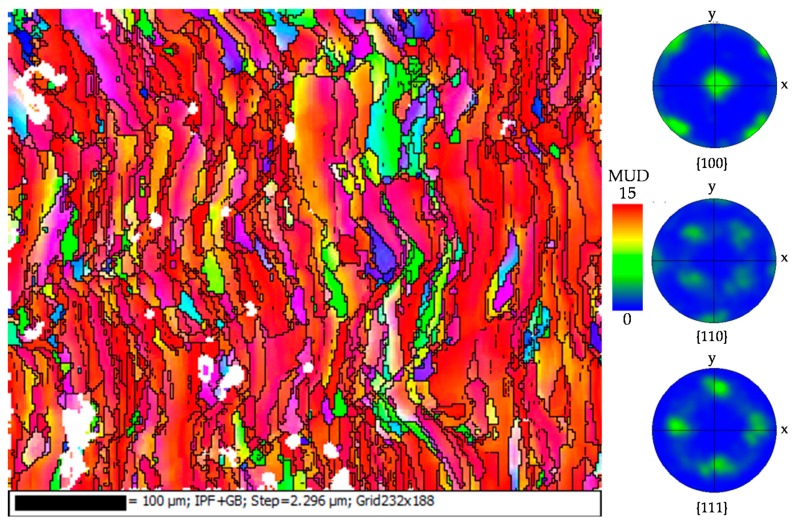
EBSD picture and pole figures for P_L_ = 400 W, v_s_ = 6500 mm/s, Δy = 20 µm, T_pre_ = 200 °C.

**Figure A4:**
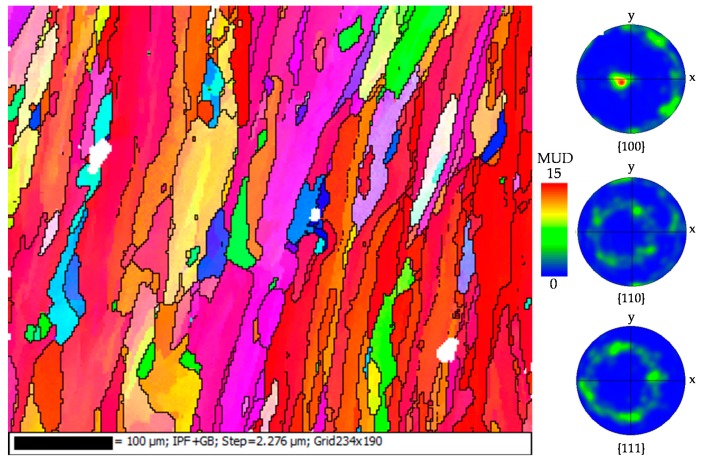
EBSD picture and pole figures for P_L_ = 200 W, v_s_ = 83 mm/s, Δy = 70 µm, T_pre_ = 3500 °C.

**Figure A5:**
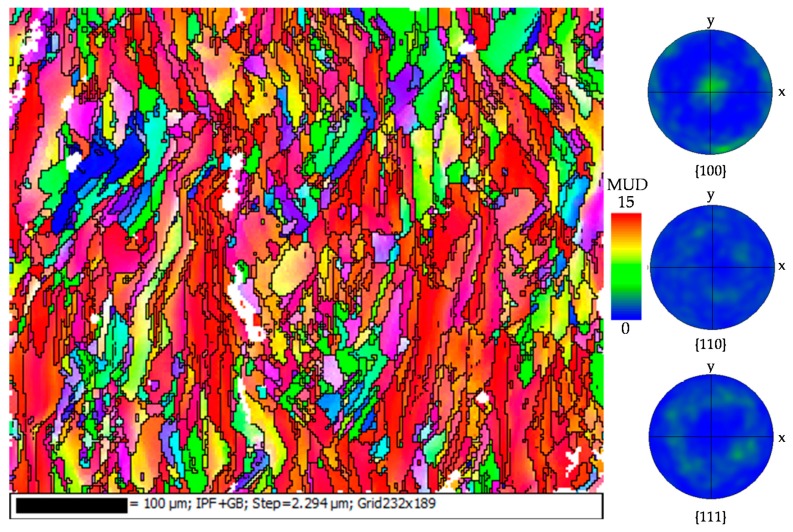
EBSD picture and pole figures for P_L_ = 300 W, v_s_ = 1240 mm/s, Δy = 78 µm, T_pre_ = 0 °C.

**Figure A6:**
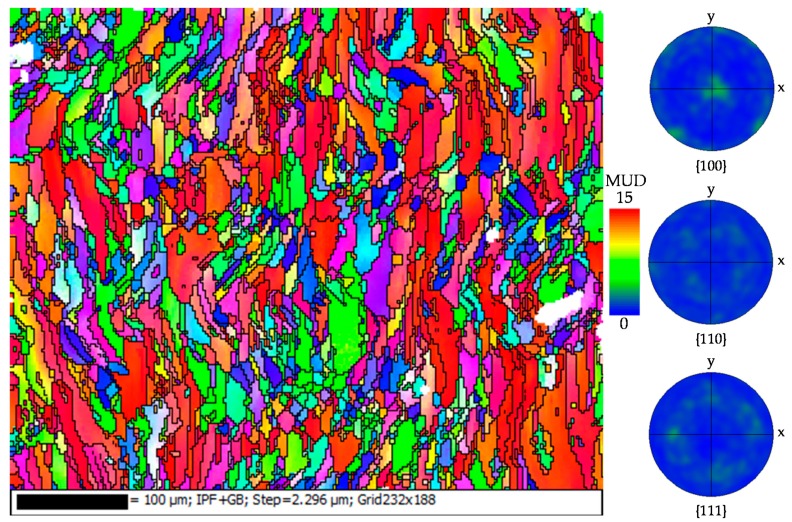
EBSD picture and pole figures for P_L_ = 400 W, v_s_ = 2820 mm/s, Δy = 83 µm, T_pre_ = 300 °C.

Figure A1 and Figure A4 are characterized by extremely elongated grains. The increased width of the grains in Figure A4 compared to Figure A1 can be attributed to lower cooling rates during processing. In both cases, grain lengths exceeding the size of the picture are visible. Figure A2 shows a grain width in the range between Figure A1 and Figure A4. However, the average grain length is significantly shorter in contrast to the other mentioned samples. A reasonable explanation for this effect might be the re-melting of lower layers during processing. By estimating the ratio d_m_/d_S_ a partial re-melting of at least 75% is assumed for Figure A1 and Figure A4 while the expected value for Figure A2 is around 50%. This leads to the assumption that the grain orientation is not only influenced by the thermal gradient in the bottom part but also at the outer boundaries of the molten pool. Large deviations between the orientation of the thermal gradient at the solidification front and the alignment of the grains lead to an interruption of the epitaxial grain growth in the early stages during solidification. Results of parameter sets depicted in Figure A3, Figure A5 and Figure A6 show a decreased grain size due to higher cooling rates. For Figure A5 and Figure A6 equiaxed grains can be found to a low extent, whereby the detectable amount is larger for Figure A6. Both parameter sets reached the columnar to equiaxed transition even though this line is not crossed completely. Analysis of set Figure A3 showed the formation of very thin and elongated grains aligned in a zig-zag orientation proving a continuous change in temperature gradients. The same effect has already been observed for PBF-EB/M by applying a uniform scanning direction for each ten consecutive layers [49]. This might be due to by the high scanning speeds causing a thermal gradient in hatch orientation perpendicular to scanning vectors and their direction. In the present case, scanning vectors are rotated by 37° after each layer while always starting at the point which is the furthest on the left of the sample. Correspondingly, the rotation of this layer is interrupted every third or fourth layer, thus being retrieved in the distance between two peaks in the zig-zag pattern. However, even though the G/R values of Figure A3 are below half of Figure A5´s almost no globular grains can be found. This might be explained by the formation and shape of the molten pool as high scanning speeds are applied during processing while the laser power is kept at a relatively low value, leading to a low line energy density. As a result, an elongated molten pool which is particularly shallow at the same time is formed, thus, supporting the epitaxial growth parallel to the building direction.

**Figure 12:**
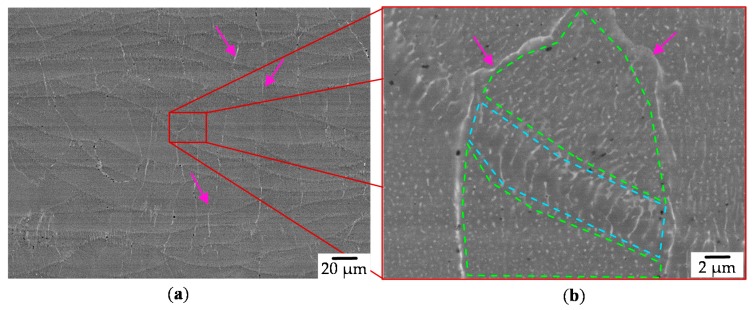
AsB Picture for sample Figure 8c. Purple arrows mark grain boundaries, blue dashed region a cellular structure at the weld seam and green most likely a degenerated columnar structures (EHT = 20 kV, WD = 8.7 mm, IProbe = 1.5 nA, ESB Grid 1000V, (**a**) Mag. 1000×, (**b**) 10.000×).

EDS and AsB measurements reveal the morphology of the grains. The corresponding grain structure for the samples is already noticeable for low magnifications (see Figure 12a). A eutectic network consisting of α-Al and θ-Al_2_Cu phase is found whereas the θ-Al_2_Cu phase is preferably formed at the grain boundaries [50]. Most likely no other phases beside the θ-Al_2_Cu phase due the rapid cooling in PBF-LB/M is formed [51]. Weld seams, which are crossing grains, are characterized by a cellular structure (Figure 12b, blue dashed region). The remaining regions of the sample show a degenerated columnar structure, as the eutectics is spread in small particles (green dashed region).

**Figure 13:**
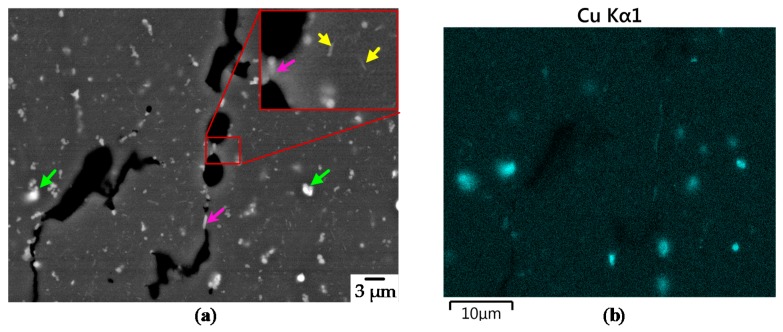
AsB Picture for sample Figure 7b. Purple arrows mark Cu rich or θ-Al_2_Cu Phase, yellow plate-shaped θ-Al_2_Cu (EHT = 20 kV, WD = 8.7 mm, IProbe = 1.5 nA, ESB Grid 1000 V).

Specimens built at T_pre_ = 350 °C show a completely different structure compared to those at T_pre_ = 20°C (see Figure 13). The grain boundaries (purple arrows) seem to degenerate and huge round Al_2_Cu/Cu particles are formed (green arrows). Instead the eutectics in the middle of the grain seems to form the typical plate-shaped θ-Al_2_Cu (yellow arrows) for temperatures above 300 °C [52].

**Figure 14:**
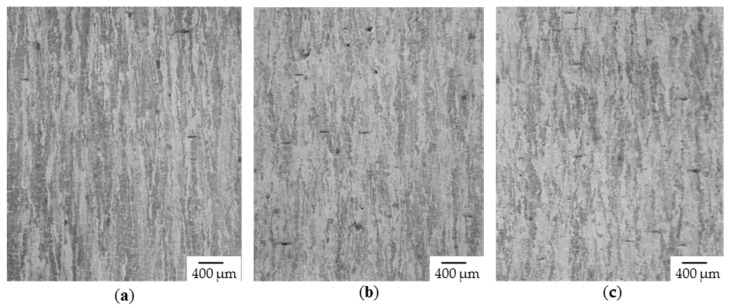
Etched cross-section (optical microcopy) with constant P_L_ = 133 W, vs = 80 mm/s, µm, T_pre_ = 20 °C (**a**) Δy = 60 µm, w_g_ = 108 µm (**b**) Δy = 70 µm, w_g_ = 103 µm (**c**) Δy = 60 µm, w_g_ = 83 µm.

Further the grain width w_g_ in dependence of the used hatch distance was investigated. It was found, that with increasing the hatch distance the mean grain width decreases (see Figure 14.) This can directly attributed to change in the cooling rate from roughly 3.5 × 10^5^ K/s to 3.8 × 10^5^ K/s.

### 3.4. Mechanical Characterization

**Figure 15:**
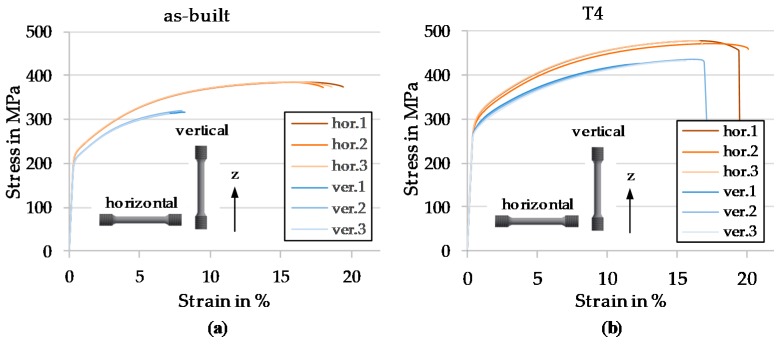
Stress strain curves (**a**) as built (**b**) T4 heat treated.

In this work six specimens were built for each build direction, vertical (v) and horizontal (h). Half of each set was T4-heat treated (see methods Section 2.5) and the remaining half was tested in as build (a. b.) condition. The stress-strain curves are show in Figure 15.

The results are summarized and compared to conventional T4 and literature in Table 6. It can be concluded that the T4 treated AM samples in both build directions exceed the values of conventionally fabricated EN AW-2024, as well as the values reported for any other PBF-LB/M sample with a chemical composition close to EN AW-2024.

**Figure 16:**
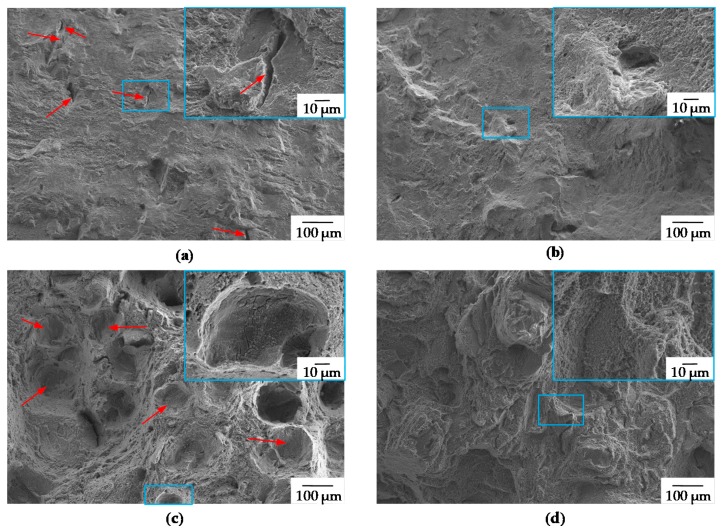
Fracture surfaces (**a**) horizontal as built, red arrows indicating cracks from insufficient binding (**b**) horizontal T4 smooth surface, typical ductile fracture surface (**c**) vertical as built, red arrows indicating “craters” corresponding to Figure 1a (**d**) vertical T4, typical ductile fracture surface. (EHT = 15 keV, WD = 13 mm, Mag = 100×/1.000×).

Taking a closer look at the stress-strain curves in Figure 15, it is obvious that samples built vertically have a reduced elongation at break and also lower values for Rp_0.2_ and R_m_. This is surprising, as those samples are characterized by highly elongated grains in the build direction, indicating less weak spots by grain boundaries. In the as-built condition the vertical samples show an even lower elongation compared to heat-treated ones. The reason for this can be found by analyzing the fracture surface. In Figure 16a, the fracture surface of an as-built horizontal tensile specimen is shown. The red arrows indicate tiny slit pores spread all over the fracture surface. In Figure 16c—as-built vertical—a bunch of cavities, also indicated using red arrows, where most likely delamination occurred, are visible. Therefore, it can be concluded that an ultra-thin layer, which is not always completely remolten by subsequent exposure, might grow on top of the weld seams during PBF. However, subsequent heat treatment appear to heal these defective layers resulting in a completely ductile fracture surface for both vertically and horizontally built samples (see Figure 16b,d).

## 4. Conclusions

In this study the effect of different solidification conditions on the resulting grain structure and morphology while processing EN AW-2024 was investigated. Samples consisting of columnar, cellular and almost complete globular grain structure could be obtained by varying the process parameters without changing the alloy composition or adding grain refiners. A simple simulation setup was already sufficient to predict the resulting grain structure beside extreme high scanning speeds. Optimized process parameters allowed for the fabrication of specimens with relative densities > 99% characterized by a negligible residual porosity and the absence of hot cracks. Finally, tensile samples have been manufactured showing the highest ever reported mechanical properties (Rp_0.2_ = 295 MPa, R_m_ = 478 MPa, A= 18,3%) for unmodified EN AW-2024 in PBF-LB/M.

## Figures and Tables

**Table 1:** Mechanical properties of EN AW-2024 in different heat treatment conditions [16].

Mechanical Properties	O	T3	T4	T62
Yield Strength (MPa)	<140	>290	>275	345
UTS (MPa)	<220	>435	>425	435
A (%)	>13	>14	>13	5

**Table 2:** Chemical composition of raw material, powder and PBF-LB/EN AW-2024 specimen.

Material	Si	Fe	Cu	Mn	Mg	Cr	Zn	Ti	Other	Al
EN AW-2024 [36]	0.5	0.5	3.8–4.9	0.3–0.9	1.2–1.8	0.1	0.25	0.15	á 0.05, tot. 0.15	balance
Raw powder	0.17	0.1	3.78	0.66	1.33	0.00	0.02	0.00	-	balance
PBF-LB/EN AW-2024	0.12	0.08	3.82	0.63	1.25	0.00	0.00	0.00	-	balance

**Table 3:** Varied parameters in the first evolvement step.

Set	P_L_ (W)	v_s_ (mm/s)	Δy (µm)	d_S_ (µm)	T_pre_ (°C)	G (K/m)	R (m/s)	GxR (K/s)	G/R (K·s/m²)
1	200	83–180	70	40	20	∙∙∙	∙∙	∙∙	∙∙∙
2	200	83–200	70	40	350	∙	∙	∙	∙
3	300, 400	580–1240	76–170	50	20	∙∙∙∙	∙∙∙	∙∙∙∙	∙∙
4	400	1560–2940	47–159	50	300	∙∙	∙∙∙∙	∙∙∙	∙

**Table 4:** Varied parameters in the second evolvement step.

Set	P_L_ (W)	v_s_ (mm/s)	Δy (µm)	d_S_ (µm)	T_pre_ (°C)	G (K/m)	R (m/s)	GxR (K/s)	G/R (K·s/m²)
5	400	3500–9000	20–60	20	200	∙∙∙∙	∙∙∙∙	∙∙∙∙	∙∙∙
6	100, 133, 166	20–160	60–170	20	20	∙∙∙	∙∙	∙∙∙	∙∙∙

**Table 5:** Calculated solidification conditions of the specimens from Section 3.1.

Sample	P_L_ W	v_s_ mm/s	Δy µm	T_pre_ °C	d_m,ex_ µm	d_m,sim_ µm	R_L_ _-_	G K/m	R m/s	GxR K/s	G/R K·s/m²
A1	133	80	60	20	76	75	0.70	1.1 × 10^7^	3.1 × 10^−2^	3.5 × 10^5^	3.6 × 10^8^
A2	200	83	70	20	86	89	0.74	9.6 × 10^6^	3.8 × 10^−2^	3.6 × 10^5^	2.6 × 10^8^
A3	400	6500	200	200	27	27	0.87	6.2 × 10^6^	2.7	1.6 × 10^7^	2.3 × 10^6^
A4	200	83	70	350	-	268	0.74	4.1 × 10^5^	4.3 × 10^−2^	1.8 × 10^4^	9.7 × 10^6^
A5	300	1240	78	20	53	55	0.86	4.6 × 10^6^	1.0	4.7 × 10^6^	4.5 × 10^6^
A6	400	2820	83	300	56	58	0.87	1.3 × 10^6^	1.8	2.3 × 10^6^	6.9 × 10^5^

**Table 6:** Results from mechanical characterization.

Property	Present Work				Zhang [15]	Wang [22]		Wrought [16]
Orientation	hor.	ver.	hor.	ver.	hor.	ver.	ver.	-
Heat-treatment	a. b.	a. b.	T4	T4	a. b.	a. b.	T6	T4
Chem. in range	yes				no	no		yes
Rp_0.2_ in MPa	227 ± 1	212 ± 0	295 ± 3	277 ± 5	276.2 ± 41	223 ± 4	368 ± 6	>275
R_m_ in MPa	387 ± 1	318 ± 2	478 ± 3	433 ± 4	402.4 ± 9.5	366 ± 7	455 ± 10	>425
A in %	18.1 ± 0.7	7.3 ± 6	18.3 ± 1.7	14.6 ± 2.6	6 ± 1.4	5.3 ± 0.3	6.2 ± 1.8	>14
